# Notes and News

**Published:** 1905-05-20

**Authors:** 


### Notes and News.

A large number of Boards of Guardians have already met to discuss the new order of the Local Government Board in regard to underfed children. In the majority of cases the matter has been referred to a committee, and it is evident the difficulties attending the subject have not been under-estimated.

The extension of the Oldham Infirmary, which forms a memorial to Queen Victoria, consists of an addition of 43 beds for patients and eight for the staff, and two new operating theatres with adjuncts. The sanitary arrangements are on the best models, and many improvements in connection with the whole building have been effected. The old operating theatres will be utilised as small wards.

A new hospital has been opened at West Hartlepool, entitled the Cameron Memorial Hospital. It has been erected by the widow, sister, and brother of the late Col. J. Cameron. It has provision for 25 adult patients, and contains one cot. The out-patients' department is extensive. £20,000 has been provided by the donors for site, building, furnishing, and establishment.

The Chorlton Guardians made an inquiry recently of the Local Government Board, as to the report of Dominion Immigration inspectors upon the children sent to Canada in 1903 and 1904. The Local Government Board could only reply that the report for 1903 was expected shortly! A great deal of interest attaches to the experimental period of the movement which is an attempt to improve and save the youthful members of the community from evil and poverty-stricken surroundings, and mould them into healthy and useful citizens. It is a pity, therefore, that a closer touch with the children has not been kept by those who sent them out. Confidence and interest are both destroyed when it is realised how long it is before information is forthcoming.

A new out-patient department has been added to the Leicester Infirmary.

A new operating theatre has been presented to the Exeter Hospital by Mrs. Nosworthy, who has contributed £1,500 for the purpose.

Typhoid fever has broken out at Fulbourn Asylum, Cambridge. There are upwards of sixty cases. At Lincoln the epidemic seems practically at an end.

A house-to-house visitation at Brockley and Lewisham was organised amongst the students of Guy's Hospital on Wednesday, to appeal for funds for the institution.

Up to the present time no further case of plague has been reported from Leith. Every precaution is being taken to disinfect the premises where the affected man worked, and to watch the contacts.

The British medical men have dispersed, after their visit to Paris, in many directions. Some went to Pau, others to Geneva. In both these places they received a warm welcome from the municipalities.

Some interesting points in connection with physical deterioration were brought out at a meeting of the Royal Statistical Society on Tuesday in a paper read by Sir Francis Sharp Powell, Bart. The statistics in the paper relate largely to employés in factories.

A bottle dealer at Lambeth made a defence for non-payment by stating that business was so bad amongst doctors that his "takings were nothing to what they used to be." The judge ordered him to pay ten shillings monthly until "the doctors' business got better."

The Royal Commission on the Care and Control of the Feeble-minded having resumed their sittings, Dr. E. Beresford, the medical superintendent of the Tooting Bec Asylum, in his evidence stated that he thought that imbeciles, defectives, and the feeble-minded might be treated together in the same institution, but that he thought their detention should be permanent. He advocated the cottage home system.

A meeting was recently held at Ayr in connection with the proposed Consumption Sanatorium for Ayrshire. It has been decided to proceed with the scheme. The estimated cost per bed appears to be about £337 10s. This is a very high figure, and in consideration of the demand for sanatoria, and the call upon charity, it seems a pity that such expensive construction is deemed necessary in connection with treatment which is described as "open-air."

